# Chlorfenapyr Crystal Polymorphism and Insecticidal Activity

**DOI:** 10.1021/acs.cgd.3c01257

**Published:** 2024-01-17

**Authors:** Reese Aronin, Petr Brázda, Leilani N. Smith, Carolyn Jin Zhang, Jason B. Benedict, Zoe Y. Marr, Boris Rybtchinski, Haim Weissman, Alexander G. Shtukenberg, Bart Kahr

**Affiliations:** † Department of Chemistry and Molecular Design Institute, New York University, 29 Washington Place, New York City, New York 10003, United States; ‡ Department of Structure Analysis, Institute of Physics, Czech Academy of Sciences, Na Slovance 2/1999 Prague 8, Prague 18221, Czech Republic; § Department of Chemistry, University at Buffalo, Buffalo, New York 14260-3000, United States; ∥ Department of Molecular Chemistry and Materials Science, Weizmann Institute of Science, Rehovot 76100, Israel

## Abstract

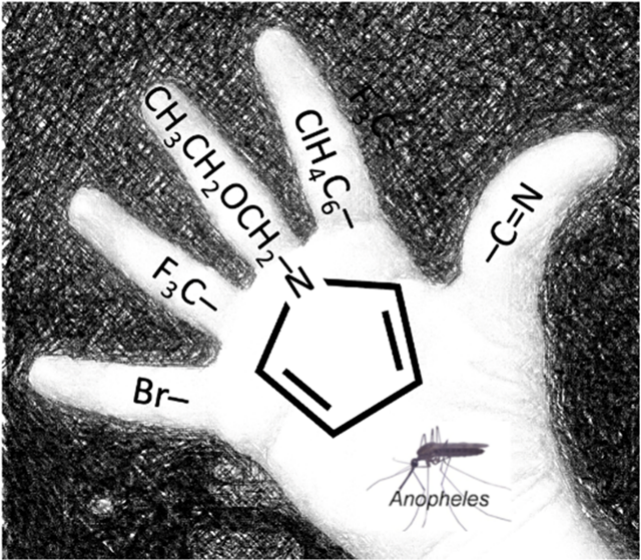

Four crystalline polymorphs of the proinsecticide chlorfenapyr [4-bromo-2-(4-chlorophenyl)-1-ethoxymethyl-5-trifluoromethyl-1*H*-pyrrole-3-carbonitrile] have been identified and characterized by polarized light optical microscopy, differential scanning calorimetry, Raman spectroscopy, X-ray diffraction, and electron diffraction. Three of the four structures were considered polytypic. Chlorfenapyr polymorphs show similar lethality against fruit flies (*Drosophila melanogaster*) and mosquitoes (*Anopheles quadrimaculatus*) with the least stable polymorph showing slightly higher lethality. Similar activities may be expected to be consistent with structural similarities. Knockdown kinetics, however, depend on an internal metabolic activating step, which further complicates polymorph-dependent bioavailability.

## Abstract 2

Four crystalline polymorphs of the proinsecticide chlorfenapyr have been identified and characterized by polarized light optical microscopy, differential scanning calorimetry, Raman spectroscopy, X-ray diffraction and electron diffraction. Three of the four structures can be considered polytypic. Chlorfenapyr crystal forms show similar lethality against fruit flies and mosquitoes with the least stable polymorph showing slightly higher lethality.

## Introduction

**Scheme 1:**
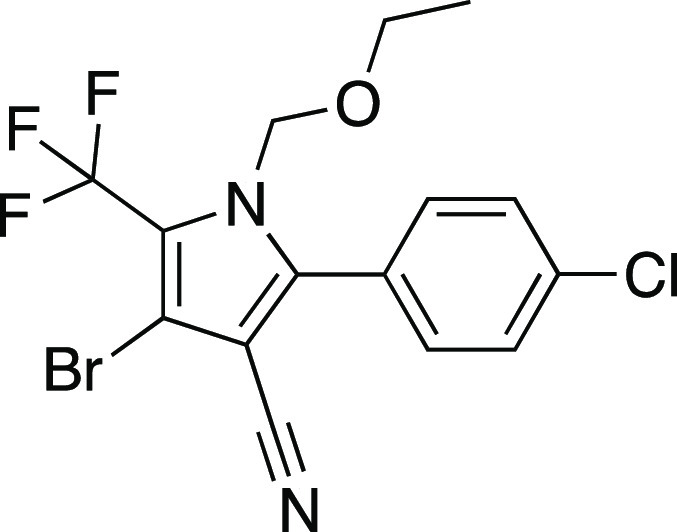
Chlorfenapyr, C_15_H_11_BrClF_3_N_2_O, *M*_w_ = 407.62 g/mol

The insecticide treadmill^1^ is the name given to the continuous search^2,3^ for toxicants with new mechanisms of action for the control of rapidly reproducing organisms that transmit disease. Malarial (*Anopheles*) mosquitoes are now widely resistant to pyrethroids, synthetic compounds related to pyrethrum, a natural insecticide found in chrysanthemums. Since the 1980s,^4^ pyrethroids have shouldered the lion’s share of malaria control. For decades, no new adulticides for vector control were approved by the World Health Organization (WHO). However, in the face of worldwide pyrethroid resistance, the WHO approved chlorfenapyr [4-bromo-2-(4-chlorophenyl)-1-ethoxymethyl-5-trifluoromethyl-1*H*-pyrrole-3-carbonitrile, CAS 122453-73-0, Scheme 1],^5^ a pyrrole proinsecticide typically used alongside pyrethroids to combat pyrethroid resistance.^6^

A proinsecticide is a compound that is metabolized to an active form inside of the target organism. Chlorfenapyr *in vivo* is subject to oxidative removal of the *N*-ethoxymethylene group. Unlike pyrethroids that target the central nervous system to prevent the closure of voltage-gated sodium ion channels,^7,8^ chlorfenapyr works by disrupting the production of ATP in the mitochondria of the target organism.^9^ Chlorfenapyr is primarily used in bed nets in combination with pyrethroids.^10,11^

The role of polymorphism in the action of pesticides and insecticides has become evident only recently.^12,13^ An inverse correlation between lethality of contact insecticides and thermodynamic stability of crystal polymorph has been found in several contact insecticides, including DDT,^14^ lindane,^15^ deltamethrin,^16,17^ and imidacloprid.^18^ The use of a more lethal polymorph of a contact insecticide can overcome resistant organisms. Greater efficiency requires the application of less active ingredient, reducing the environmental impact.

Given the increasing importance of polymorphism in the activity of contact insecticides, it is surprising that there is no information in the literature about the solid states of chlorfenapyr. This work is intended to fill this gap. Without knowledge of the landscape of polymorphs, it is uncertain whether chlorfenapyr is being used in its most active forms.

Here, four polymorphs of chlorfenapyr have been identified and characterized using polarized optical microscopy (POM), differential scanning calorimetry (DSC), Raman spectroscopy, powder X-ray diffraction (PXRD), single-crystal X-ray diffraction (SCXRD), transmission electron microscopy (TEM), and 3D electron diffraction (3D ED). From the melt, chlorfenapyr yields two polymorphs (I and IV), while from solution, chlorfenapyr crystallizes as two other distinct but structurally similar polymorphs (II and III), which are similar to form I. Bioassays performed using fruit flies and mosquitoes demonstrate similar lethality for all four forms, with least stable polymorph IV showing slightly higher lethality.

## Experimental Section

Several milligrams of chlorfenapyr (ChemCruz, Lot B2323) was melted between two cover glass slides (0.1 mm thick) using a hot stage (model FP90, Mettler Toledo) or a Kofler bench at ca. 110 °C. Within 3–5 s, the melt was cooled to the target temperature, at which crystallization occurred spontaneously. Crystallization above room temperature was performed using a hot stage, and crystallization at 10 °C was performed in a refrigerator. Crystallization and phase transformations were monitored using polarized light optical microscopes (Olympus BX50 and BX53) equipped with digital cameras. The linear growth rate of chlorfenapyr I and IV were measured as the linear advance of the growth front per unit time.

Chlorfenapyr was also crystallized by solvent evaporation at room temperature from acetone, acetic acid, tetrahydrofuran, chloroform, diethyl ether, hexanes, acetonitrile, toluene, methanol, and chlorobenzene solutions.

Chlorfenapyr II crystals were also obtained via the blooming out of poly(ethylene) fibers. High density and low density poly(ethylene) powders (Sigma-Aldrich) were mixed in a 2:1 ratio. Then, chlorfenapyr was added in a concentration of 1 wt %. Approximately 0.3 g of a mixture was melted at 130–150 °C and short sections of fibers of ca. 0.5 mm in diameter were manually pulled out of melt. Crystallization on the fiber surface started within 2 days.

Differential scanning calorimetry (DSC) was performed using a PerkinElmer DSC 8000 instrument. Chlorfenapyr was heated at a rate of 10 °C/min and cooled at 30 °C/min. An indium standard was used to calibrate the instrument, and nitrogen was used as the purge gas. The data was analyzed using the PerkinElmer software to extract the glass-transition temperature, melting points, and heats of fusion and transformation.

Raman spectra were collected with a Raman microscope (DXR, Thermo Fisher Scientific) using a 532 nm excitation laser operating at 3 mW, a full-range grating, and a 25 μm pinhole. The data were analyzed with OMNIC software.

Powder X-ray diffraction (PXRD) patterns were collected by using a Bruker D8 Discover General Area Detector Diffraction System (GADDS) equipped with a VÅNTEC-2000 2D detector and Cu-Kα source (λ = 1.54178 Å). The X-ray beam was monochromated with a graphite crystal and collimated with a 0.5 mm capillary collimator (MONOCAP). Chlorfenapyr powder was loaded into 0.8 mm Kapton capillaries, and PXRD patterns were collected in transmission mode.

The single crystal X-ray diffraction (SCXRD) data set for chlorfenapyr I was recorded on a Bruker D8 APEX-II CCD system with the ω scans at 100 K using graphite-monochromated and 0.5 mm MonoCap-collimated Mo-*K*α radiation (λ = 0.71073 Å). Structures were solved by intrinsic phasing methods (SHELXT)^19^ and the models were refined with SHELXL^20^ using least-squares minimization (full-matrix least-squares on *F*^2^). The single crystal was grown from the melt at supercooling of less than ten degrees.

The single-crystal X-ray diffraction data set for chlorfenapyr II was collected at the NSF’s ChemMatCARS Sector 15 Advanced Photon Source (APS) at Argonne National Laboratory (λ = 0.41329 Å). The single crystal (180 μm × 60 μm × 50 μm) was mounted on the tip of a glass fiber with oil and placed on a Huber three-circle diffractometer. Using a Dectris Pilatus3X 1 M (CdTe) shutterless detector at 130 mm from the crystal, frames were collected with ω = −180° and a 2θ angle of 0°. During the data collection, the crystal was cooled to 100 K by using an Oxford cryojet nitrogen gas flow apparatus. A total of 1440 frames were collected during two 360° φ-scans (0.5° image width) at κ = 0° and κ = 30°, nominally covering complete reciprocal space. Dectris frames (.cbf) were converted to Bruker format (.sfrm) using custom software developed by NSF’s ChemMatCARS. Following frame conversion, indexing was performed using the Bruker APEX3 software suite. Data reduction was completed using SAINT version 8.40A, and a multiscan absorption correction was applied using SADABS version 2016 included in the Bruker APEX3 software suite. Space group determination was performed using the XPREP utility included in the SHELXTL software package.^21^ Using Olex2,^22^ the structure was solved with SHELXT^17^ using intrinsic phasing and refined with SHELXL^18^ using least-squares minimization (full-matrix least-squares on *F*^2^).

Transmission electron microscopy (TEM) was used to search for new, metastable polymorphs. Aliquots of 50 nL of filtered chloroform solution of chlorfenapyr 0.8 w/w % was applied on a holey carbon-coated Cu TEM grid (200-mesh) via a 1 μL syringe (blotless preparation), allowed to age for 12 min at 23 °C, and then frozen in liquid nitrogen. TEM imaging was carried out at liquid nitrogen temperature on a Titan Krios G3i instrument (Thermo Fisher Scientific) operating at 300 kV. The samples were introduced into the instrument by using an autoloader. Images were recorded by using a Gatan K3 direct detection camera. The darker contrast rounded features spread over the images are due to ice contamination.

3D electron diffraction (3D ED) was performed on an FEI Tecnai G^2^ 20 microscope (200 kV, λ = 0.0251 Å) with a LaB_6_ cathode equipped with a Cheetah ASI direct detection camera (16 bit). Temperature of measurement was 150 K (sample holder tip temperature) for acetone and hexanes crystallized powder and for melt-grown material. Measurement of fiber-grown crystals was done at 185 K. Data were measured using continuous rotation electron diffraction method with integration semiangle of 0.125°, except for melt-grown material where precession-assisted electron diffraction geometry was used (precession angle was 0.8°). The material was gently ground if necessary and directly deposited on the TEM Cu grid. Data was processed with PETS2.^23^ Optical distortions were compensated using calibrated values.^24^ Structures were solved by Superflip^25^ and Sir2014.^26^ Structure models were refined in Jana2020^27^ using dynamical refinement.^28^ In case of continuous rotation data, the concept of overlapping virtual frames was used.^29^

Crystallographic information files (CIFs), including the HKL and RES data, for all crystal structures were deposited at the Cambridge Crystallographic Data Centre (CCDC); the corresponding CCDC numbers are indicated in Table 1.

**Table 1:** Crystallography of Chlorfenapyr Polymorphs

	form I	form II	form II	form II	form III	form IV
crystallization method	melt	acetone solution	exsolution from PE fiber	methanol solution	hexanes solution	melt
structure solution	SCXRD	3D ED^a^	3D ED^b^	SCXRD	3D ED	3D ED^a^
*T*, K	296	150	185	100	150	150
space group, CIF file^c^	*Pca*2_1_	*Pa*	*Pa*	*Pc*	*Pcab*	*P*2_1_/*n*
*a*, Å^c^	17.224(4)	8.078(1)	8.18(1)	22.767(4)	8.056(1)	12.259(1)
*b*, Å^c^	12.049(3)	17.460(2)	17.35(2)	17.484(3)	17.414(1)	15.453(1)
*c*, Å^c^	7.9527(18)	22.707(6)	23.1(7)	8.0290(12)	45.406(5)	8.583(1)
β, °^c^	90	91.53(1)	92(1)	91.166(3)	90	104.14(1)
space group, same setting^d^	*P*2_1_*ab*	*Pa*	*Pa*	*Pa*	*Pcab*	*P*2_1_/*n*
*a*, Å^d^	7.9527(18)	8.078(1)	8.18(1)	8.0290(12)	8.056(1)	12.259(1)
*b*, Å^d^	17.224(4)	17.460(2)	17.35(2)	17.484(3)	17.414(1)	15.453(1)
*c*, Å^d^	12.049(3)	22.707(6)	23.1(7)	22.767(4)	45.406(5)	8.583(1)
β, °^d^	90	91.53(1)	92(1)	91.166(3)	90	104.14(1)
cell volume, Å^3^	1650.4(7)	3201.5	3276	3195.3(8)	6369.9	1576.6
*Z*, *Z*′	4, 1	8, 4	8, 4	8, 4	16, 2	4, 1
number of reflections	2916	21304	7582	13777	19856	20460
number of parameters	238	499	378	834	258	338
Flack parameter	0.09(3)			0.039(17)		
*R*^e^	0.0439	0.1151	0.1552	0.0684	0.1044	0.0820
*R*_w_^f^	0.1185	0.1246	0.1273	0.1950	0.1121	0.0891
*GoF*	1.053	3.549	3.220	1.030	1.793	1.452
CCDC number	2297417	2297498	2297499	2292170	2297500	2297501

a Combination of four data sets.

b Combination of two data sets.

c Space groups and lattice constants are shown in the same form as in the CIF files deposited to the CCDC.

d For better comparison for forms I, II, and III, space groups and lattice constants are converted to the same setting.

e *I* > 2σ_*I*_ for SCXRD data, and *I* > 3σ_*I*_ for 3D ED data.

f *w* = 1/[σ^2^(*F*_o_^2^) + (*k*_1_*P*)^2^ + *k*_2_*P*], where *P* = (*F*_o_^2^ + 2*F*_c_^2^)/3 for SCXRD data with *k*_1_ = 0.0529 and *k*_2_ = 0.8843 for form I, and *k*_1_ = 0.0973 and *k*_2_ = 14.0430 for form II, respectively. *w* = 1/[σ^2^(*F*) + 0.0001*F*^2^] for all 3D ED data except form II from acetone for which *w* = 1/[σ^2^(F_o_) + (0.01*P*)^2^], where *P* = (*F*_o_^2^ + 2*F*_c_^2^)/3.

The lethality of chlorfenapyr polymorphs was determined through unlimited exposure bioassays against fruit flies, **Drosophila melanogaster**, and mosquitoes, *Anopheles quadrimaculatus*. Lethality measurements were conducted in triplicate with 10 insects per replicate. Experiments 3 and 4 for form I and experiments 3, 4, and 5 for form III had only two replicates. Fruit flies and mosquitoes were sedated with CO_2_ and then transferred to 30 mm or 60 mm Fisher polystyrene Petri dishes, respectively, for holding to ensure all insects were alive and conscious before exposure. For *D*. *melanogaster*, 3 mg of powders of each polymorph was uniformly spread over 60 mm diameter Pyrex borosilicate glass Petri dishes. Forms I and III were ground before weighing, and all dishes were shaken for easy dispersion. Since *A. quadrimaculatus* are larger and more fragile than *D*. *melanogaster*, 3 mg of each polymorph were dispersed in 100 mm diameter glass Petri dishes. Insects were then transferred from their respective holding containers to their glass Petri dish counterpart. Knockdown measurements as prescribed by WHO were used every 5 min to report lethality until all insects were rendered in the supine position.

## Polymorphism

**Figure 1:**
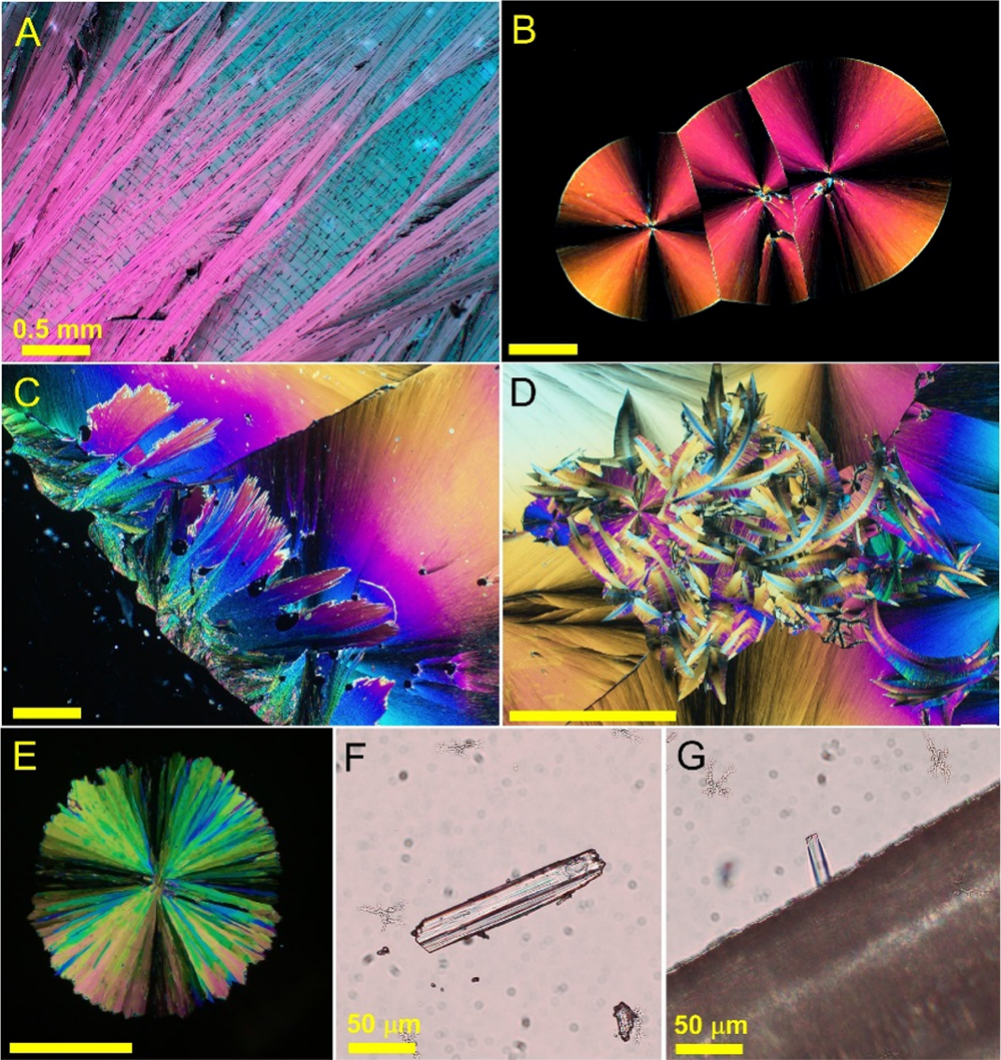
Optical micrographs of chlorfenapyr polymorphs. Polarizers are crossed for images (A–E). (A) Form I was crystallized from the melt at 80 °C. (B) Form I crystallized from the melt at 23 °C. (C) Transformation of form I into form II/III starting from the edge of the slide at the lower left corner. (D) Feathery flakes of form IV grown from the melt and surrounded by spherulites of form I. (E) Spherulite form IV nucleated at 50 °C. (F) Crystal of form III grown from hexanes. (G) Needle of form II formed on the surface of a poly(ethylene) fiber. If not specified, the scale bars are 0.5 mm.

There are no known crystal structures of chlorfenapyr in the open literature. Some derivatives have been published, however.^30,31^ Chlorfenapyr crystallization from the melt is dominated by form I in the whole range of growth temperatures between 10 °C and a melting point, *T*_m_ = 101.4 °C, heat of fusion 21.8 kJ/mol. Close to the melting point, it forms needlelike crystals, which are replaced by coarse, optically negative spherulites around 80 °C (Figure 1A). The spherulites become finer at lower temperatures (Figure 1B). Chlorfenapyr I is a thermodynamically stable polymorph above 59.5 °C. Below that temperature, it becomes metastable and slowly (on a scale of months between glass slides) converts to form II or III (Figure 1C; because of the small amount of material and structure similarities, it was not possible to identify which polymorph it was). Forms II and III do not directly nucleate from the melt but readily crystallize from solutions.

**Figure 2:**
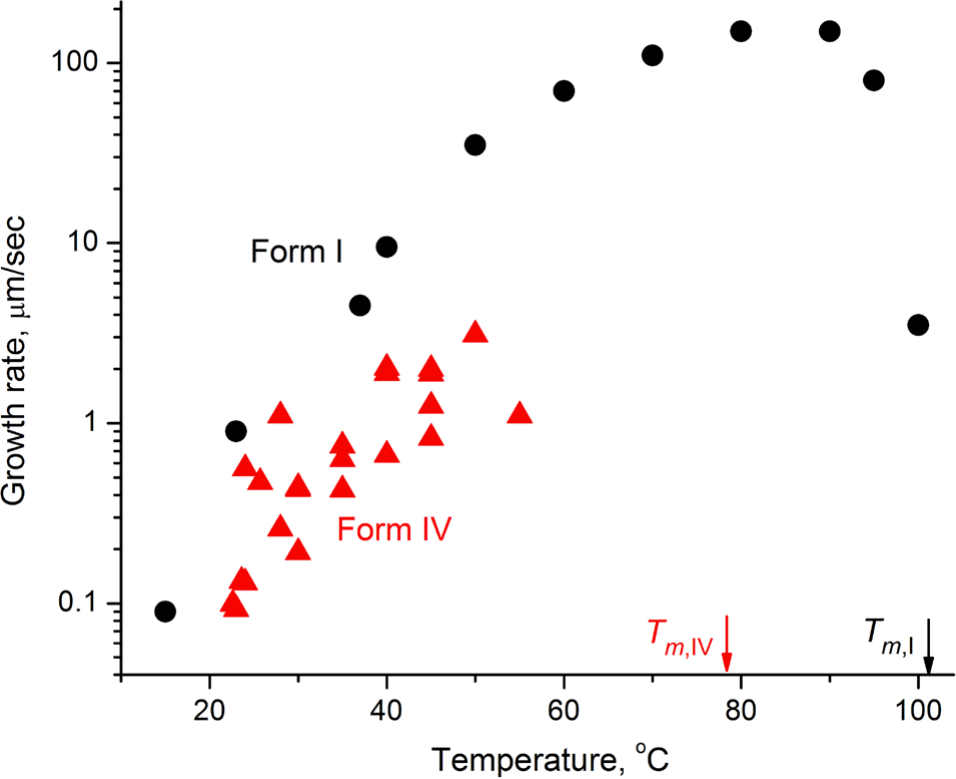
Linear growth rates of chlorfenapyr I (black circles) and IV (red triangles) from the melt. Significant scattering of points for form IV is related to its growth anisotropy.

**Figure 3:**
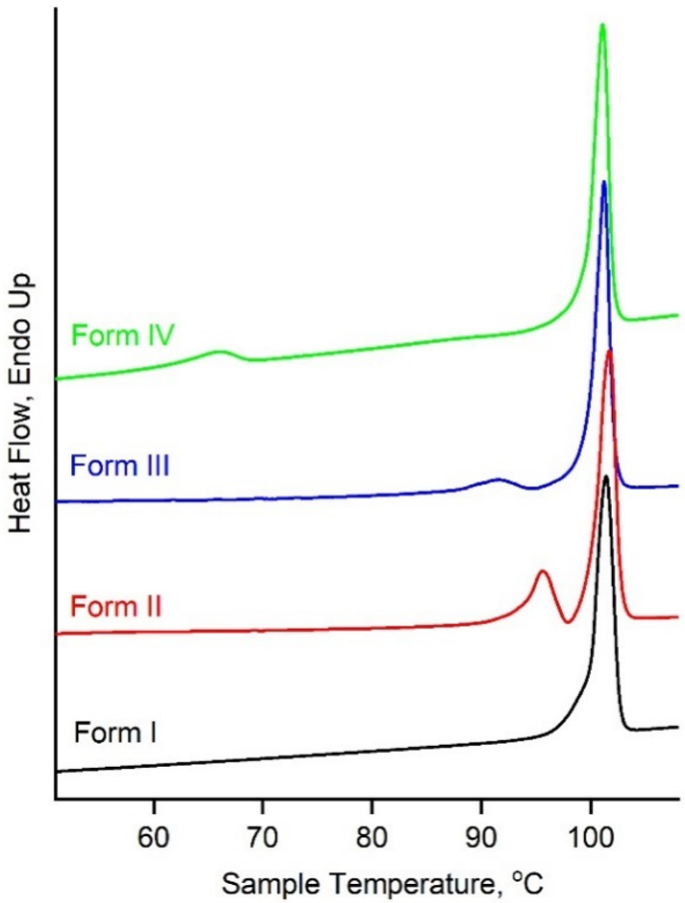
DSC heating curves for chlorfenapyr polymorphs. Heating rate is 10 °C/min. Endotherms around 101 °C correspond to melting of form I. Endotherms around 88–96 °C correspond to II → I and III → I phase transformations and are likely to partial melting of forms II and III. An endotherm around 61–68 °C corresponds to IV → I phase transformation.

**Figure 4:**
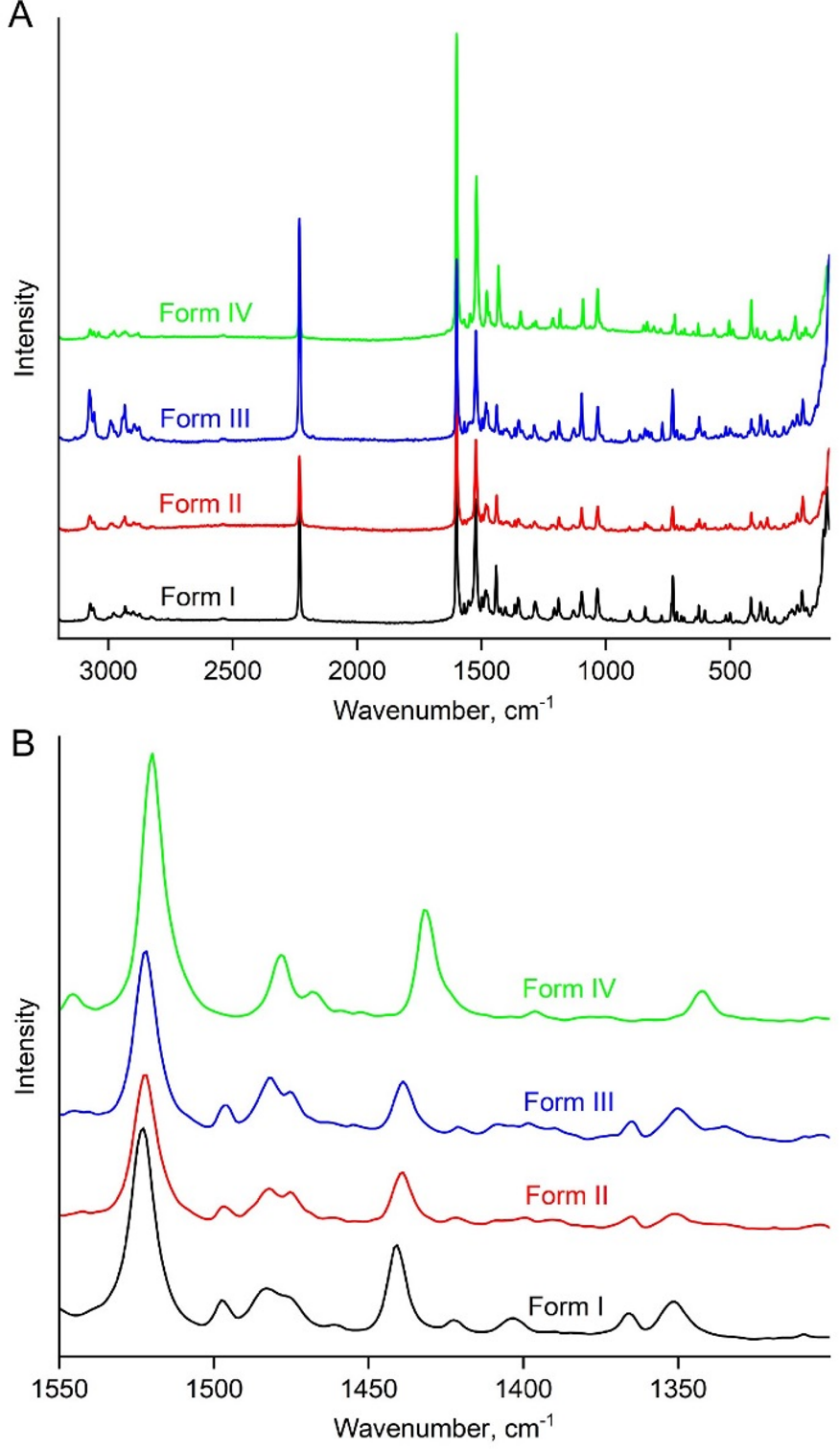
Raman spectra of chlorfenapyr polymorphs (A) and a fragment highlighting the differences between polymorphs (B).

**Figure 5:**
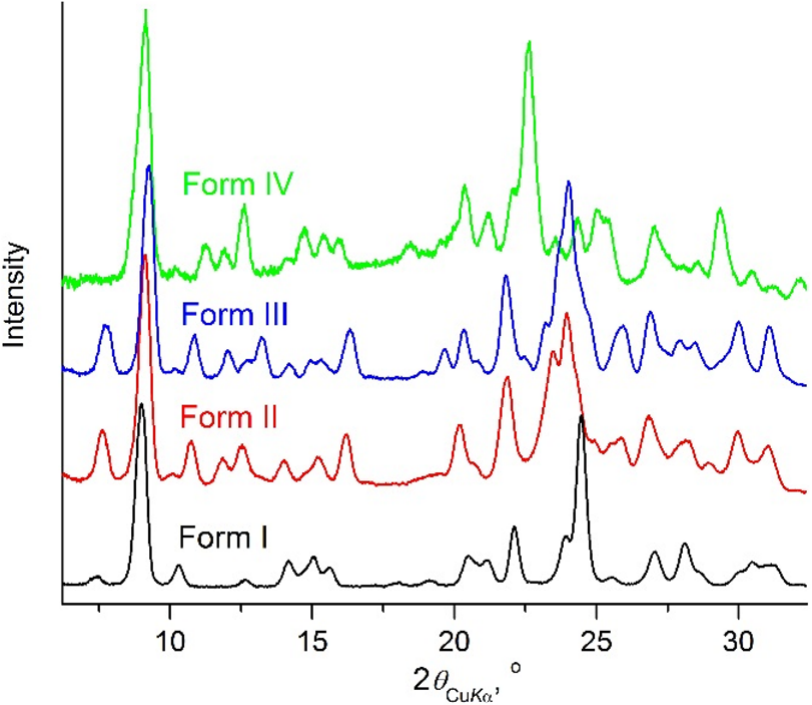
Powder X-ray diffraction patterns for chlorfenapyr polymorphs. Forms I and IV were crystallized from the melt, while forms II and III were obtained from acetone and hexanes solutions, respectively.

From the melt, below ca. 50 °C, chlorfenapyr I is accompanied by a small fraction of form IV (*T*_m_ = 78 °C, Figure 1E), which forms coarse spherulites above 30 °C and appears as curved feathery flakes at lower temperatures (Figure 1D). A small fraction of form IV is related not only to smaller nucleation rates but also to smaller growth rates, which show significant anisotropy (Figure 2). Such curved morphologies with anisotropic growth rates are common for crystallization slightly (up to a few tens of degrees) above glass-transition temperature (*T*_g_ = −15.3 °C) and signal onset of glass-to-crystal growth mode with anomalous rate acceleration.^32,33^ Form IV is the least stable polymorph of chlorfenapyr, which at room temperature converts to form I within 1–2 months between glass slides and within hours in a powdery form. Transformation takes only several seconds above 60 °C, which prevents measurement of the melting point and heat of fusion directly by DSC (Figure 3). The heat of transformation IV → I is 2.2 kJ/mol. Both polymorphs crystallizing from the melt exhibit distinct Raman spectra (Figure 4) and PXRD patterns (Figure 5).

Solution crystallization from all solvents used in this study resulted in a family of polymorphs that exhibit similar morphologies (Figure 1F), almost identical Raman spectra (Figure 4), similar PXRD patterns (Figure 5) and melting points, and *T*_m_ = 93–96 °C, thereby suggesting substantial similarities in their crystal structures. Obtaining melting points and heats of fusion was not possible from DSC data because these polymorphs rapidly convert to form I above 85–90 °C (Figure 3). PXRD patterns facilitated the identification of two distinct polymorphs, II and III, whose crystal structures were solved using electron diffraction. Form II was obtained from methanol, acetone, acetic acid, toluene, tetrahydrofuran, chloroform, chlorobenzene, acetonitrile, and diethyl ether, while form III was prepared from toluene, acetone, and hexanes, as well as mixtures of methanol and acetonitrile. Commercial chlorfenapyr was identified as form III. It is worth noting that the patterns assigned to the same polymorph were not always identical but showed minor variations in peak intensities and positions, which can be related to variations in the polymorphic composition. Also, crystallization from the same solvent, such as acetone or toluene, can produce different polymorphs depending on the growth conditions. For example, as confirmed by PXRD, crystallization from acetone at high evaporation rate (crystallization took about 10 min) resulted in form II, while slow evaporation (crystallization took 6 h) provided form III.

**Figure 6:**
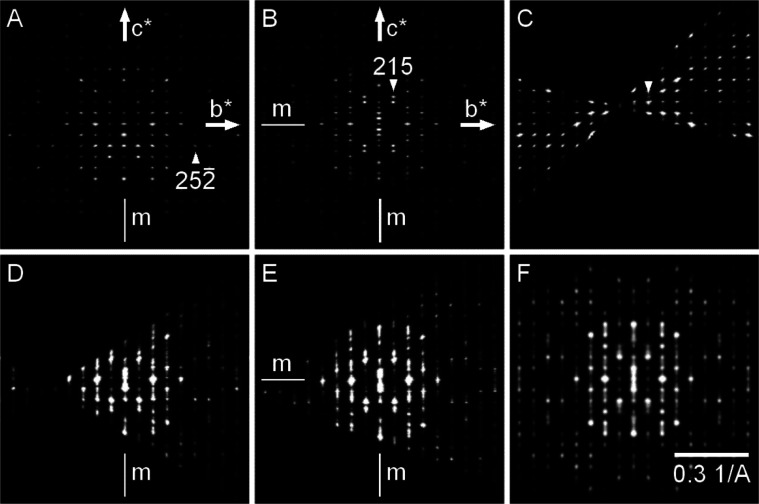
*2kl* reciprocal space sections of 3D ED data from chlorfenapyr II and III. (A) Ordered form II, (B) ordered form III, (C) probable intergrowth of form II (large domain) and form III (small domain, arrow indicates the strongest reflection of form III with odd *l* index), (D) disordered form II where symmetry of the intensities follows the monoclinic symmetry (only vertical mirror symmetry), (E) disordered form II where symmetry of the intensities corresponds to orthorhombic symmetry (both vertical and horizontal mirror symmetry), and (F) simulation of (E) using 1:1 combination of smeared form II and III *2kl* sections with induced orthorhombic symmetry.

A very similar competing formation of forms II and III was observed in 3D ED. Materials crystallized from acetone, hexanes, and chloroform were studied. Chlorfenapyr prepared from acetone showed intense diffuse scattering, thereby suggesting frequent layer stacking faults along c*. The major phase was form II, but form III crystallites were also found, although often disordered. Finding a form II crystallite without diffuse scattering was very difficult (Figure 6A). Material crystallized from hexanes showed improved crystallinity, and the major phase was form III. Finding a well-ordered form III was relatively easy (Figure 6B). A probable intergrowth of forms II and III where the domain of form III was much smaller was also found (Figure 6C). Particles prepared by crystallization from chloroform were the least ordered, and all showed significant diffuse scattering.

These observations were similar to the crystals blooming on the polyethylene fibers. It was often impossible to define periodicity along c* for crystals grown on the fibers, but the β angle was close to the monoclinic ordered form II. Interestingly, there were crystals where the monoclinic symmetry of the diffracted intensities was still relatively well preserved (Figure 6D), while others had a symmetry resembling that of the orthorhombic form III (Figure 6E). We tried to simulate the data by smearing the reciprocal space sections of forms II and III along the c* direction to get the narrowing of the coherently diffracting domains due to the stacking faults along this direction and by combining the diffraction patterns of the two forms. This is an approximation, which omits the diffracted intensity coming from the volume close to the domain boundaries. The best match between data (Figure 6E) and this approximation was found for a 1:1 combination of the form II and III smeared patterns (Figure 6F). Thus, form II crystallized on poly(ethylene) fiber surfaces because of the blooming process (Figure 1G) is largely disordered and probably contains small domains of form III. This experiment mimics chlorfenapyr blooming in poly(ethylene) insecticidal bednets, the process that has been already simulated experimentally but for which no crystallographic information has been obtained.^34,35^

**Figure 7:**
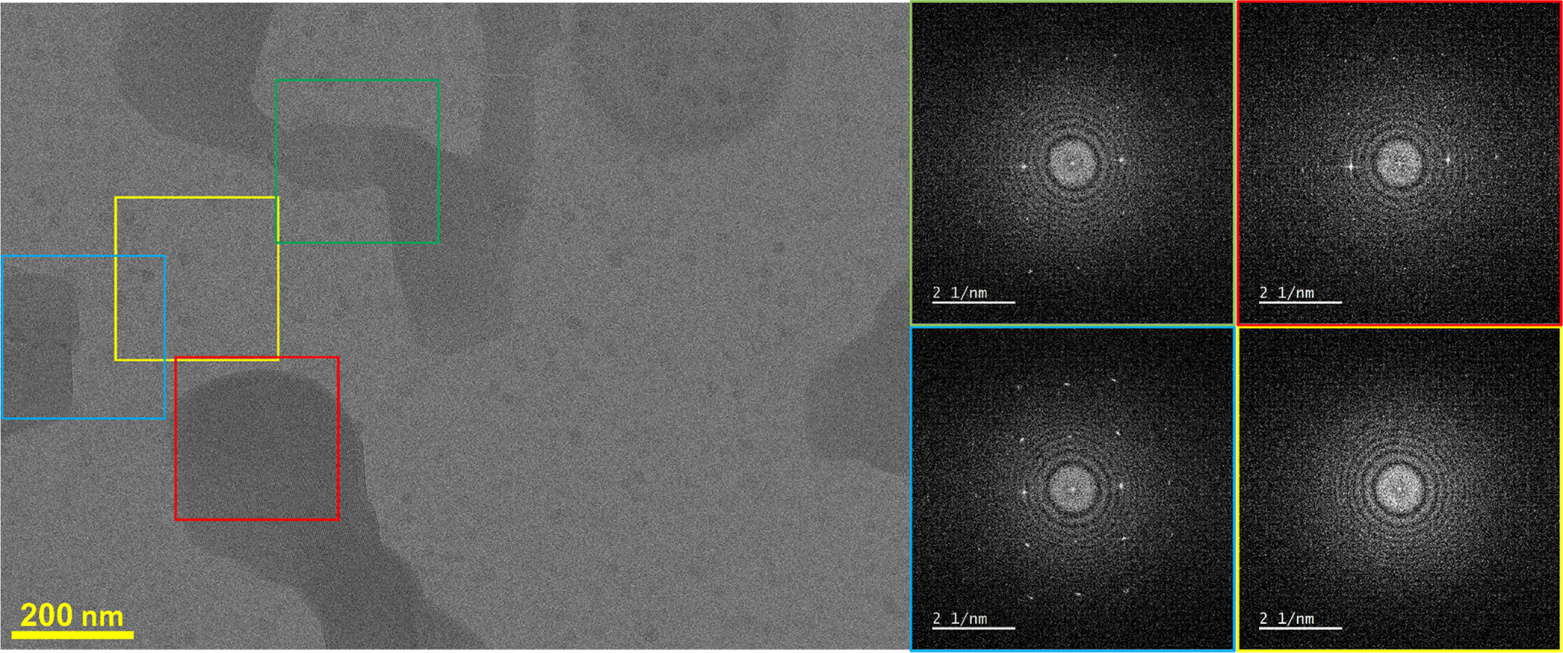
TEM image of chlorfenapyr deposited from 0.8 wt % chloroform solution on a holey carbon grid and kept at room temperature for 12 min. The imaging was performed under cryogenic conditions. The areas marked with different colors correspond to the fast Fourier transforms (FFTs) marked with the same color. The boxed areas (blue, red, and green) present crystalline domains with FFT patterns that correspond to spacings of 4.46 ± 0.02 and 3.48 ± 0.02 Å. The area marked in yellow is vitrified ice without organic material showing no FFT pattern of chlorfenapyr. The blue area corresponds to a single crystal of form II, and the green and blue correspond to a mixture of forms II and III.

TEM imaging of chlorfenapyr deposited from chloroform revealed formation of a mixture of forms II and III, as evidenced by fast Fourier transforms (FFTs) (Figure 7) that correspond well to the diffraction of forms II and III (Figure 6). This confirms a trend to crystallize in a mixture of these forms also from solution.

## Crystal Structures

**Figure 8:**
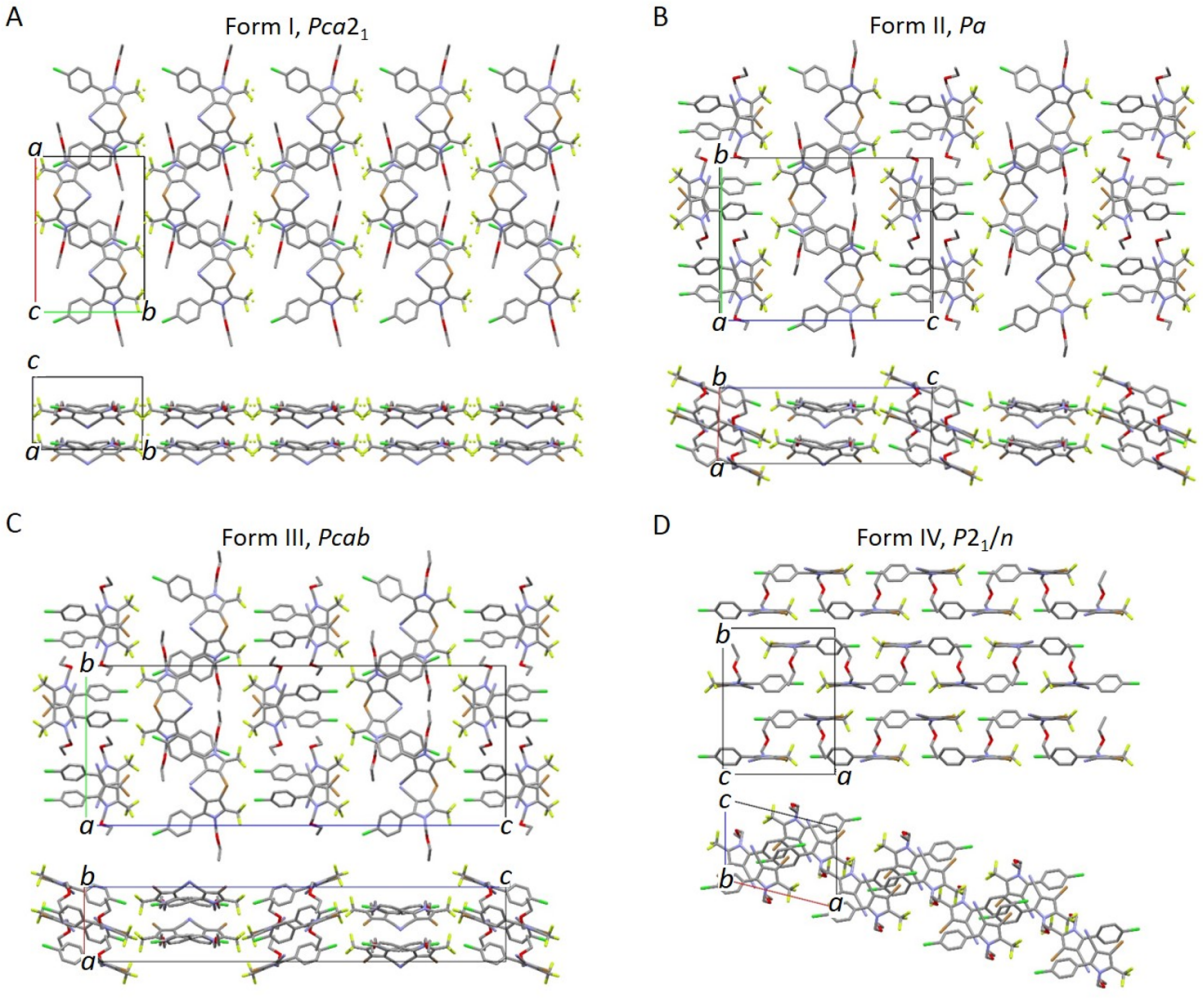
Crystal structures of chlorfenapyr polymorphs. Hydrogen atoms have been omitted for clarity.

Crystal structures of all four polymorphs are summarized in Table 1 and are shown in Figure 8. Polymorphs I, II, and III are organized in a similar way and can be described as family of polytypes (polymorphs that consist of same types of layers but differ mainly by their stacking sequences). They consist of parallel sheets in which chlorfenapyr molecules are oriented roughly perpendicular to the sheet surface so that the sheets are bound by the halogen atoms F, Cl, and Br. Within a sheet, molecules either form two populations with Cl ends directed toward opposite surfaces of the sheet (A type), or all molecules have their Cl ends directed toward the same surface of the sheet (B type). Polymorph I contains only A sheets in the same orientation so that the sheet sequence can be depicted as AAAAA... (Figure 8A). Polymorph II has two sheets in the same orientation, and the sheet sequence is ABABAB... (Figure 8B). Polymorph III contains the same two types of sheets, but their orientations are alternating by 180° rotation along the *c*-axis to make a motif ABA′B′ABA′B′ABA′B′... (Figure 8C). Given the similarities of the crystal structures of chlorfenapyr II and III, some disordered sheet sequences and more complex polytypes are also expected but were not confirmed in this study. The minor differences observed in PXRD patterns for material crystallized from different solvents possibly result from such disorder. All chlorfenapyr molecules in the crystal structure of form IV are also roughly parallel to one another; however, they are displaced along the *a*-axis so that no sheets perpendicular to their elongation direction are formed (Figure 8D).

## Insecticidal activity

**Figure 9:**
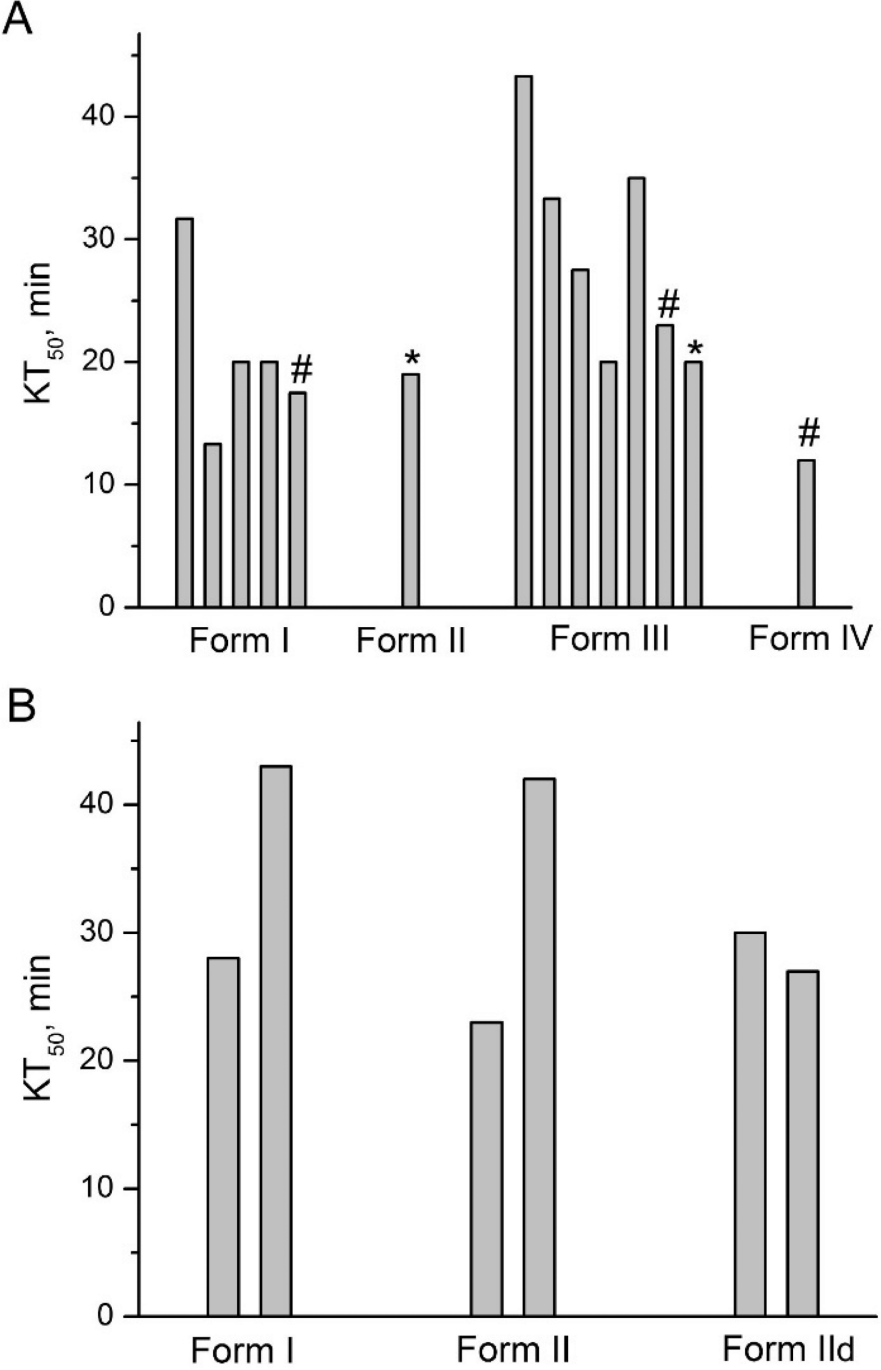
KT_50_ obtained for chlorfenapyr I and IV crystallized from the melt, chlorfenapyr II crystallized from acetone, chlorfenapyr IId crystallized from chloroform, and chlorfenapyr III as commercial form. Different bars correspond to different trials. (A) Fruit flies, *Drosophila melanogaster*. Bars marked with * correspond to the bioassay performed on the same day for which chlorfenapyr II and III were crystallized from acetone solution. Bars marked with # correspond to bioassay performed on the same day. (B) *A. quadrimaculatus* mosquitoes.

Lethality of chlorfenapyr polymorphs was tested against fruit flies and mosquitoes (Figure 9). Chlorfenapyr I and IV were crystallized from the melt. Chlorfenapyr II was prepared from acetone solution, and its disordered version IId was prepared from chloroform solutions. Commercial material was used as chlorfenapyr III, and in one bioassay, this polymorph was obtained from acetone. In general, the median knockdown time (KT_50_) values are similar for different polymorphs and show significant variability for different trials, which can be related to a more complex mode of action of chlorfenapyr, which requires additional metabolic steps. Forms I, II, IId, and III are very similar structurally (see above), and there is no surprise that they show similar lethality. Samples of forms II and III prepared from acetone and tested on the same day (trials marked with * in Figure 9A) showed very similar lethality. Chlorfenapyr IV has a distinct crystal structure, and on the basis of its low melting point, it is characterized by the highest free energy. This polymorph shows slightly higher lethality, which agrees with previous observations on other insecticides.^12−14,16^ Because of the small fraction of this polymorph in the melt-grown samples, however, we were able to perform only one bioassay (trials on different polymorphs performed simultaneously are marked with # in Figure 9A) and could not estimate how statistically significant this effect is.

## Conclusions

Four crystalline polymorphs of the proinsecticide chlorfenapyr have been identified and characterized by polarized light optical microscopy, differential scanning calorimetry, and Raman spectroscopy. Their crystal structures were solved using single-crystal X-ray diffraction and 3D electron diffraction. Three of the four structures can be considered polytypic. Because of disorder, severe twinning, and small size, successful crystal structure determination for forms II, III, and IV, to a great extent, was achieved by the electron diffraction method.

Chlorfenapyr polymorphs show similar lethality against fruit flies (*Drosophila melanogaster*) and mosquitoes (*Anopheles quadrimaculatus*), with the least stable polymorph showing slightly higher lethality. This is the first example for which strong correlation between polymorph free energy difference and insect lethality has not been detected; however, in the case of chlorfenapyr, the difference between three of four crystals are minimal, and knockdown kinetics is more complicated since it depends on internal metabolic activating step.
